# Evaluation of HPV Knowledge Levels Among Healthcare Professionals and Medical Students: A Multicenter Cross‐Sectional Study in Private Hospitals and Foundation Universities in Turkey

**DOI:** 10.1155/ogi/3367094

**Published:** 2026-07-22

**Authors:** Inci Öz, Batu Öz, Asena Ayar Madenli, Gürkan Kıran

**Affiliations:** ^1^ Department of Gynaecology of Obstetrics, Istinye University Faculty of Medicine, Istanbul, Turkey; ^2^ Department of Gynaecology of Obstetrics, Medicana Atakoy Hospital, Istanbul, Turkey; ^3^ Bezmialem University Faculty of Medicine, Istanbul, Turkey, bezmialem.edu.tr; ^4^ Department of Gynaecology of Obstetrics, Bezmialem University Faculty of Medicine, Istanbul, Turkey, bezmialem.edu.tr

**Keywords:** cervical cancer prevention, healthcare professionals, HPV vaccination, human papillomavirus, knowledge assessment, medical students, public health education, screening programs

## Abstract

**Objectives:**

To assess the knowledge and attitudes toward human papillomavirus (HPV) and HPV vaccination among healthcare professionals and medical students and to identify demographic and professional factors associated with variations in HPV knowledge.

**Methods:**

This cross‐sectional study included 903 participants comprising medical doctors, medical students, nurses, and other allied healthcare personnel. HPV knowledge was measured using the validated 33‐item Human Papillomavirus Knowledge Scale (HPV‐KS), which evaluates four domains: general HPV knowledge, HPV screening tests, HPV vaccination, and the national HPV vaccination program. Demographic, educational, occupational, and geographical variables were also collected. Total and domain‐specific scores were compared across gender, professional groups, and educational levels.

**Results:**

The mean total HPV knowledge score was 19.23 out of 33, indicating moderate overall knowledge. Correct response rates were high for core factual items (e.g., HPV as the leading cause of cervical cancer), yet several misconceptions persisted, including misunderstandings about HIV/AIDS causation and HPV transmission dynamics. Male participants demonstrated significantly higher total knowledge (*p* = 0.008) and greater HPV screening test knowledge (*p* = 0.003) than females. Professional category did not yield significant differences in total scores although medical doctors and medical students showed slightly higher vaccination‐related knowledge. Participants who were not physicians or medical students had higher screening and vaccination knowledge, whereas physicians and medical students scored higher on vaccination program knowledge.

**Conclusions:**

HPV knowledge among healthcare professionals and medical students was moderate, with persistent misconceptions across several domains. Gender‐based differences and unexpected patterns among physicians and medical students suggest that existing curricula may not adequately address HPV and HPV vaccination. Strengthening formal education, integrating evidence‐based HPV modules into health sciences training, and providing structured professional development are essential to enhancing HPV awareness, supporting vaccination advocacy, and improving preventive health practices.

## 1. Introduction

Human papillomavirus (HPV) is the most common sexually transmitted infection worldwide and a central etiological factor in the development of cervical cancer. Persistent infection with high‐risk HPV subtypes is responsible for the vast majority of cervical malignancies, making HPV prevention and vaccination a major global public health priority. Serrano et al. emphasize that HPV is so widespread that most sexually active individuals are infected at some point during their lives [1]. Despite the availability of safe and effective vaccines, cervical cancer continues to contribute substantially to morbidity and mortality, particularly in settings where screening and vaccination uptake remain suboptimal [2].

Healthcare professionals and medical students play a pivotal role in HPV prevention, patient education, vaccination advocacy, and adherence to screening guidelines [3]. Their level of knowledge directly influences public awareness and vaccine acceptance. However, multiple investigations demonstrate that healthcare trainees and even practicing clinicians frequently harbor significant knowledge gaps and misconceptions concerning HPV transmission, disease burden, and immunization protocols. Solis‐Torres et al. evaluated a sample of medical students in Puerto Rico and demonstrated important knowledge gaps regarding HPV, HPV vaccination, and associated cancers [4]. Similarly, a study among university students found inadequate understanding of HPV infection and prevention strategies, even within educated young adult populations [5].

These gaps are not limited to students. Shakurnia et al. showed that although the majority of healthcare providers had adequate HPV‐related knowledge, important deficiencies and low vaccination uptake persisted among them, indicating an ongoing need for targeted education [6]. Such deficiencies pose a barrier to the effective implementation of HPV vaccination programs and may limit healthcare professionals’ ability to provide evidence‐based guidance. Effective clinical communication about HPV vaccination has been shown to increase vaccine acceptance, underscoring the importance of provider education and confidence in discussing HPV‐related topics.

In this context, evaluating awareness and knowledge among current and future healthcare providers is critically important. The present study adds to the existing literature by examining HPV knowledge across a large, diverse cohort of healthcare professionals and medical students.

## 2. Materials and Methods

### 2.1. Ethical Consideration

Ethical approval was granted by the Bezmialem University Ethics Committee (approval date: June 5, 2025; decision number: 2025/165). All study procedures adhered to the ethical standards outlined in the Declaration of Helsinki.

### 2.2. Study Design and Participants

This cross‐sectional analytical study evaluated the knowledge and attitudes regarding HPV among healthcare professionals and medical students. A total of 903 participants were included. Eligible individuals were aged 18 years or older, involved in healthcare education or clinical practice, and able to complete the survey in Turkish. Participation was voluntary and anonymous.

### 2.3. Survey Instrument

HPV knowledge was assessed using the 33‐item Human Papillomavirus Knowledge Scale (HPV‐KS) [7]. The instrument consists of true/false items and includes four domains (1): General HPV Knowledge (Items 1–16), (2) HPV Screening Test Knowledge (Items 17–22), (3) HPV Vaccine Knowledge (Items 23–27), and (4) Knowledge of the HPV National Vaccination Program (Items 28–33). Correct responses received 1 point; incorrect or “I do not know” responses received 0 points. Total scores ranged from 0 to 33, with higher scores reflecting greater knowledge.

### 2.4. Additional Variables

The questionnaire collected demographic and contextual information, including age, gender, professional category, highest educational level, nationality, academic status, and whether the participant was a physician or a medical student. These variables were used for subgroup comparisons.

### 2.5. Data Collection Procedures

Data were collected via an online self‐administered survey distributed through institutional networks, academic mailing lists, and healthcare communication platforms. The survey could be completed on mobile or desktop devices. Incomplete, duplicate, or inconsistent responses were excluded before analysis.

### 2.6. Statistical Analyses and Tools

Statistical analyses were conducted incorporating Python‐based libraries (SciPy, Scikit‐learn, and Statsmodels). Data distribution was assessed via skewness, kurtosis, and the Shapiro–Wilk test. Comparative analyses employed chi‐square or Fisher’s exact tests for categorical variables, and Kruskal–Wallis, one‐way ANOVA, *t*‐test, or Mann–Whitney *U* tests for categorical–numerical comparisons. Pearson and Spearman correlations were used for numerical associations. A *p* value < 0.05 was considered significant.

## 3. Results

### 3.1. Participant Characteristics

**FIGURE 1:**
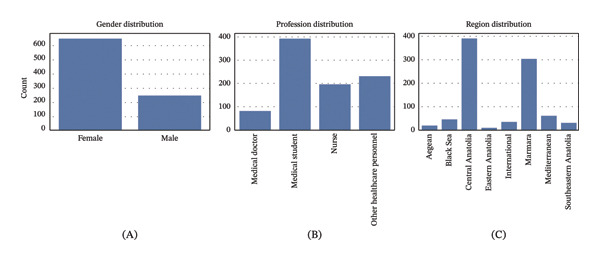
Baseline distribution of demographic and occupational characteristics among participants. (A) Distribution of participants by gender (female vs. male). (B) Distribution of professional categories, including medical doctors, medical students, nurses, and other healthcare personnel. (C) Geographic distribution of participants across eight regions (Aegean, Black Sea, Central Anatolia, Eastern Anatolia, International participants, Marmara, Mediterranean, and Southeastern Anatolia).

A total of 903 participants were included in the study. Of these, 72.8% were female and 27.2% were male. The mean age was 26.03 years. Medical students constituted the largest group (43.5%), followed by nurses (21.5%), other healthcare personnel (25.7%), and medical doctors (9.3%). More than half of the participants (52.8%) were physicians or medical students, and the majority resided in the Central Anatolia (43.4%) and Marmara (33.7%) regions. Most participants were Turkish nationals (96.1%), and 91.6% had a university‐level education (Table 1). A detailed visual summary of gender, professional distribution, and geographic representation is provided in Figure 1.

**TABLE 1:** Baseline characteristics of all participants.

	Mean, *N* (%)
*N*	903 (100%)
Gender	
Female	657 (72.8%)
Male	246 (27.2%)
Total	903 (100%)
Age	26.03
Professional category	
Medical doctor	84 (9.3%)
Medical student	393 (43.5%)
Nurse	194 (21.5%)
Other healthcare personnel	232 (25.7%)
Total	903 (100%)
Physician or medical student	
No	426 (47.2%)
Yes	477 (52.8%)
Total	903 (100%)
Region	
Aegean Region	20 (2.2%)
Black Sea Region	46 (5.1%)
Central Anatolia Region	392 (43.4%)
Eastern Anatolia Region	11 (1.2%)
International	35 (3.9%)
Marmara Region	304 (33.7%)
Mediterranean Region	63 (7.0%)
Southeastern Anatolia Region	32 (3.5%)
Total	903 (100%)
Nationality	
International	35 (3.9%)
National	868 (96.1%)
Total	903 (100%)
Education	
Academician	54 (6.0%)
Nontertiary educated	76 (8.4%)
University	773 (85.6%)
Total	903 (100%)
University education	
No	76 (8.4%)
Yes	827 (91.6%)
Total	903 (100%)

### 3.2. Overall HPV Knowledge Performance

**FIGURE 2:**
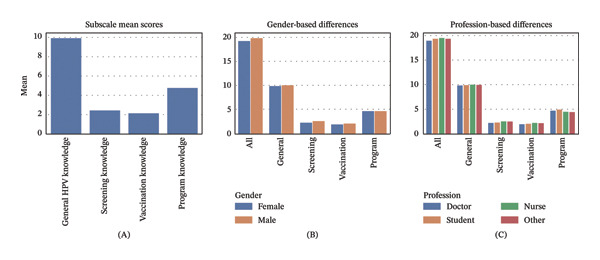
Comparison of HPV knowledge components across key demographic factors. (A) Mean scores of four HPV knowledge subscales: general HPV knowledge, screening test knowledge, vaccination knowledge, and knowledge of the national HPV vaccination program. (B) HPV knowledge differences between female and male participants across all subscales. (C) Subscale score differences across professional categories (medical doctors, medical students, nurses, and other healthcare personnel).

The mean total HPV knowledge score was 19.23 out of 33. Subscale means were as follows: General HPV Knowledge 9.92, HPV Screening Test Knowledge 2.44, HPV Vaccination Knowledge 2.12, and Knowledge of the Current HPV Vaccination Program 4.74. Correct response rates were high for core factual items such as the association between HPV and cervical cancer (98.1%) and sexual transmission (94.0%). Persistent misconceptions were noted, including beliefs that HPV causes HIV/AIDS (45.3%) or that most individuals will not acquire HPV during their lifetime (56.0%). Awareness of vaccination schedules was mixed; whereas 86.7% knew the three‐dose requirement, and 28.7% incorrectly assumed the vaccine is licensed for women aged 30–45 years (Table 2). Subscale performance and demographic differences in HPV knowledge across gender and professional groups are illustrated in Figure 2.

**TABLE 2:** Participants’ responses to the questionnaire items, including correct and incorrect answer rates.

	Mean ‐ *N* (%)
All True Total Mean	19.23
General HPV Knowledge True Total Mean	9.92
Knowledge of HPV Screening Tests True Total Mean	2.44
Knowledge of HPV Vaccination True Total Mean	2.12
Knowledge of the Current HPV Vaccination Program True Total Mean	4.74
(1) HPV can cause cervical cancer.	
False	17 (1.9%)
True	886 (98.1%)
Total	903 (100%)
(2) A person could have HPV for many years without knowing it.	
False	69 (7.6%)
True	834 (92.4%)
Total	903 (100%)
(3) Having many sexual partners increases the risk of getting HPV.	
False	24 (2.7%)
True	879 (97.3%)
Total	903 (100%)
(4) HPV is very rare.	
False	778 (86.3%)
True	123 (13.7%)
Total	901 (100%)
(5) HPV can be passed on during sexual intercourse.	
False	54 (6.0%)
True	849 (94.0%)
Total	903 (100%)
(6) HPV always has visible signs or symptoms.	
False	746 (82.6%)
True	157 (17.4%)
Total	903 (100%)
(7) Using condoms reduces the risk of getting HPV.	
False	118 (13.1%)
True	780 (86.9%)
Total	898 (100%)
(8) HPV can cause HIV/AIDS.	
False	409 (45.3%)
True	494 (54.7%)
Total	903 (100%)
(9) HPV can be passed on by genital skin‐to‐skin contact.	
False	149 (16.5%)
True	754 (83.5%)
Total	903 (100%)
(10) Men cannot get HPV.	
False	789 (87.4%)
True	114 (12.6%)
Total	903 (100%)
(11) Having sex at an early age increases the risk of getting HPV.	
False	250 (27.7%)
True	653 (72.3%)
Total	903 (100%)
(12) There are many types of HPV.	
False	59 (6.6%)
True	841 (93.4%)
Total	900 (100%)
(13) HPV can cause genital warts.	
False	61 (6.8%)
True	842 (93.2%)
Total	903 (100%)
(14) HPV can be cured with antibiotics.	
False	708 (78.5%)
True	194 (21.5%)
Total	902 (100%)
(15) Most sexually active people will get HPV at some point in their lives.	
False	506 (56.0%)
True	397 (44.0%)
Total	903 (100%)
(16) HPV usually does not need any treatment.	
False	739 (81.8%)
True	164 (18.2%)
Total	903 (100%)
(17) If a woman tests positive for HPV, she will definitely get cervical cancer.	
False	778 (86.2%)
True	125 (13.8%)
Total	903 (100%)
(18) An HPV test can be done at the same time as a Pap test.	
False	161 (17.8%)
True	742 (82.2%)
Total	903 (100%)
(19) An HPV test can tell you how long you have had an HPV infection.	
False	689 (76.5%)
True	212 (23.5%)
Total	901 (100%)
(20) HPV testing is used to indicate if the HPV vaccine is needed.	
False	639 (70.8%)
True	263 (29.2%)
Total	902 (100%)
(21) When you have an HPV test, you get the results the same day.	
False	531 (58.9%)
True	371 (41.1%)
Total	902 (100%)
(22) If an HPV test shows that a woman does not have HPV, her risk of cervical cancer is low.	
False	408 (45.2%)
True	494 (54.8%)
Total	902 (100%)
(23) Girls who have had an HPV vaccine do not need a Pap test when they are older.	
False	780 (86.4%)
True	123 (13.6%)
Total	903 (100%)
(24) One of the HPV vaccines offers protection against genital warts.	
False	223 (24.7%)
True	680 (75.3%)
Total	903 (100%)
(25) The HPV vaccines offer protection against all sexually transmitted infections.	
False	710 (78.6%)
True	193 (21.4%)
Total	903 (100%)
(26) Someone who has an HPV vaccine cannot develop cervical cancer.	
False	739 (81.8%)
True	164 (18.2%)
Total	903 (100%)
(27) HPV vaccines offer protection against most cervical cancers.	
False	143 (15.9%)
True	758 (84.1%)
Total	901 (100%)
(28) The HPV vaccine requires three doses.	
False	120 (13.3%)
True	782 (86.7%)
Total	902 (100%)
(29) The HPV vaccines are most effective if given to people who have never had sex.	
False	297 (33.0%)
True	604 (67.0%)
Total	901 (100%)
(30) HPV vaccine is recommended for all females aged 11–26 years.	
False	124 (13.7%)
True	778 (86.3%)
Total	902 (100%)
(31) HPV vaccine is licensed for women aged 30–45 years.	
False	259 (28.7%)
True	642 (71.3%)
Total	901 (100%)
(32) Both HPV vaccines that are available (Gardasil and Cervarix) protect against both genital warts and cervical cancer.	
False	77 (8.5%)
True	826 (91.5%)
Total	903 (100%)
(33) HPV vaccine is permitted for males aged 11–26 years.	
False	253 (28.0%)
True	650 (72.0%)
Total	903 (100%)

### 3.3. Gender‐Based Differences

Male participants had slightly higher total knowledge scores compared with females (19.68 vs. 19.07, *p* = 0.008). Men also demonstrated significantly higher knowledge of HPV screening tests (2.65 vs. 2.37, *p* = 0.003). No significant gender differences were observed in general HPV knowledge, vaccination knowledge, or vaccination prograknowledge (Table 3) (Figure 2).

**TABLE 3:** Comparative analysis of HPV questionnaire results across gender groups.

	Female	Male	*p*
	Mean (%)	Mean (%)	
*N*	657 (72.8%)	246 (27.2%)	
Age	26.19	25.59	0.463
Professional category			
Medical doctor	53 (8.1%)	31 (12.6%)	**0.002**
Medical student	269 (40.9%)	124 (50.4%)	
Nurse	153 (23.3%)	41 (16.7%)	
Other healthcare personnel	182 (27.7%)	50 (20.3%)	
Total	657 (100%)	246 (100%)	
Physician or medical student			
No	335 (51.0%)	91 (37.0%)	**< 0.001**
Yes	322 (49.0%)	155 (63.0%)	
Total	657 (100%)	246 (100%)	
Region			
Aegean Region	17 (2.6%)	3 (1.2%)	0.251
Black Sea Region	37 (5.6%)	9 (3.7%)	
Central Anatolia Region	283 (43.1%)	109 (44.3%)	
Eastern Anatolia Region	9 (1.4%)	2 (0.8%)	
International	26 (4.0%)	9 (3.7%)	
Marmara Region	209 (31.8%)	95 (38.6%)	
Mediterranean Region	52 (7.9%)	11 (4.5%)	
Southeastern Anatolia Region	24 (3.7%)	8 (3.3%)	
Total	657 (100%)	246 (100%)	
Education			
Academician	33 (5.0%)	21 (8.5%)	0.104
Nontertiary educated	53 (8.1%)	23 (9.3%)	
University	571 (86.9%)	202 (82.1%)	
Total	657 (100%)	246 (100%)	
All True Total	19.07	19.68	**0.008**
General HPV Knowledge True Total	9.88	10.05	0.066
Knowledge of HPV Screening Tests True Total	2.37	2.65	**0.003**
Knowledge of HPV Vaccination True Total	2.09	2.20	0.230
Knowledge of the Current HPV Vaccination Program True Total	4.73	4.78	0.636

*Note:* Bold values represent variables showing statistically significant differences between female and male participants (*p* < 0.05).

### 3.4. Differences by Professional Category

Age differed significantly across professional categories, with medical doctors representing the oldest group and medical students the youngest (Table 4). Participants who were physicians or medical students were predominantly younger. HPV screening test knowledge (*p* = 0.016) and HPV vaccination knowledge (*p* = 0.014) were higher among participants who were not physicians or medical students, whereas physicians and medical students scored significantly higher on vaccination program knowledge (*p* < 0.001) (Table 5).

**TABLE 4:** Comparative analysis of HPV questionnaire results across professional category groups.

	Medical doctor	Medical student	Nurse	Other healthcare personnel	*p*
	Mean (%)	Mean (%)	Mean (%)	Mean (%)	
Gender					
Female	53 (63.1%)	269 (68.4%)	153 (78.9%)	182 (78.4%)	**0.002**
Male	31 (36.9%)	124 (31.6%)	41 (21.1%)	50 (21.6%)	
Total	84 (100%)	393 (100%)	194 (100%)	232 (100%)	
Age	31.88	23.06	27.16	27.99	**< 0.001**
Physician or medical student					
No	0 (0.0%)	0 (0.0%)	194 (100.0%)	232 (100.0%)	**< 0.001**
Yes	84 (100.0%)	393 (100.0%)	0 (0.0%)	0 (0.0%)	
Total	84 (100%)	393 (100%)	194 (100%)	232 (100%)	
Region					
Aegean Region	3 (3.6%)	8 (2.0%)	2 (1.0%)	7 (3.0%)	**< 0.001**
Black Sea Region	1 (1.2%)	11 (2.8%)	19 (9.8%)	15 (6.5%)	
Central Anatolia Region	41 (48.8%)	122 (31.0%)	113 (58.2%)	116 (50.0%)	
Eastern Anatolia Region	3 (3.6%)	0 (0.0%)	6 (3.1%)	2 (0.9%)	
International	12 (14.3%)	14 (3.6%)	2 (1.0%)	7 (3.0%)	
Marmara Region	13 (15.5%)	209 (53.2%)	24 (12.4%)	58 (25.0%)	
Mediterranean Region	6 (7.1%)	18 (4.6%)	14 (7.2%)	25 (10.8%)	
Southeastern Anatolia Region	5 (6.0%)	11 (2.8%)	14 (7.2%)	2 (0.9%)	
Total	84 (100%)	393 (100%)	194 (100%)	232 (100%)	
Education					
Academician	43 (51.2%)	0 (0.0%)	8 (4.1%)	3 (1.3%)	**< 0.001**
Nontertiary educated	1 (1.2%)	3 (0.8%)	23 (11.9%)	49 (21.1%)	
University	40 (47.6%)	390 (99.2%)	163 (84.0%)	180 (77.6%)	
Total	84 (100%)	393 (100%)	194 (100%)	232 (100%)	
All True Total	18.85	19.25	19.37	19.22	0.248
General HPV Knowledge True Total	9.86	9.91	9.96	9.94	0.987
Knowledge of HPV Screening Tests True Total	2.27	2.32	2.59	2.59	0.098
Knowledge of HPV Vaccination True Total	1.99	2.03	2.27	2.22	0.099
Knowledge of the Current HPV Vaccination Program True Total	4.73	5.00	4.55	4.48	**< 0.001**

*Note:* Boldface indicates statistically significant *p* values (*p* < 0.05) in comparisons among the four professional category groups.

**TABLE 5:** Comparative analysis of HPV questionnaire results by physician or medical student status.

	No	Yes	*p*
	Mean (%)	Mean (%)	
Gender			
Female	335 (78.6%)	322 (67.5%)	**< 0.001**
Male	91 (21.4%)	155 (32.5%)	
Total	426 (100%)	477 (100%)	
Age	27.61	24.61	**< 0.001**
Professional category			
Medical doctor	0 (0.0%)	84 (17.6%)	
Medical student	0 (0.0%)	393 (82.4%)	
Nurse	194 (45.5%)	0 (0.0%)	
Other healthcare personnel	232 (54.5%)	0 (0.0%)	
Total	426 (100%)	477 (100%)	
Region			
Aegean Region	9 (2.1%)	11 (2.3%)	**< 0.001**
Black Sea Region	34 (8.0%)	12 (2.5%)	
Central Anatolia Region	229 (53.8%)	163 (34.2%)	
Eastern Anatolia Region	8 (1.9%)	3 (0.6%)	
International	9 (2.1%)	26 (5.5%)	
Marmara Region	82 (19.2%)	222 (46.5%)	
Mediterranean Region	39 (9.2%)	24 (5.0%)	
Southeastern Anatolia Region	16 (3.8%)	16 (3.4%)	
Total	426 (100%)	477 (100%)	
Education			
Academician	11 (2.6%)	43 (9.0%)	**< 0.001**
Nontertiary educated	72 (16.9%)	4 (0.8%)	
University	343 (80.5%)	430 (90.1%)	
Total	426 (100%)	477 (100%)	
All True Total	19.29	19.18	0.338
General HPV Knowledge True Total	9.95	9.90	0.898
Knowledge of HPV Screening Tests True Total	2.59	2.31	**0.016**
Knowledge of HPV Vaccination True Total	2.24	2.02	**0.014**
Knowledge of the Current HPV Vaccination Program True Total	4.51	4.95	**< 0.001**

*Note:* Bold values indicate statistically significant differences between participants who were physicians or medical students and those who were not (*p* < 0.05).

### 3.5. Differences by Educational Level

Academicians were older and predominantly medical doctors, while nontertiary educated individuals were more frequently other healthcare personnel. University‐educated individuals formed the majority of the sample. HPV vaccination knowledge differed significantly across education levels (*p* = 0.018), with the nontertiary educated group demonstrating the highest mean score in this subscale. Total knowledge scores did not differ significantly between educational categories (Table 6).

**TABLE 6:** Comparative analysis of HPV questionnaire results across education status groups.

	Academician	Nontertiary educated	University	*p*
	Mean (%)	Mean (%)	Mean (%)	
Gender				
Female	33 (61.1%)	53 (69.7%)	571 (73.9%)	0.104
Male	21 (38.9%)	23 (30.3%)	202 (26.1%)	
Total	54 (100%)	76 (100%)	773 (100%)	
Age	32.57	31.63	25.02	**< 0.001**
Professional category				
Medical doctor	43 (79.6%)	1 (1.3%)	40 (5.2%)	**< 0.001**
Medical student	0 (0.0%)	3 (3.9%)	390 (50.5%)	
Nurse	8 (14.8%)	23 (30.3%)	163 (21.1%)	
Other healthcare personnel	3 (5.6%)	49 (64.5%)	180 (23.3%)	
Total	54 (100%)	76 (100%)	773 (100%)	
Physician or medical student				
No	11 (20.4%)	72 (94.7%)	343 (44.4%)	**< 0.001**
Yes	43 (79.6%)	4 (5.3%)	430 (55.6%)	
Total	54 (100%)	76 (100%)	773 (100%)	
Region				
Aegean Region	0 (0.0%)	1 (1.3%)	19 (2.5%)	**< 0.001**
Black Sea Region	1 (1.9%)	5 (6.6%)	40 (5.2%)	
Central Anatolia Region	31 (57.4%)	52 (68.4%)	309 (40.0%)	
Eastern Anatolia Region	1 (1.9%)	1 (1.3%)	9 (1.2%)	
International	7 (13.0%)	0 (0.0%)	28 (3.6%)	
Marmara Region	9 (16.7%)	9 (11.8%)	286 (37.0%)	
Mediterranean Region	2 (3.7%)	8 (10.5%)	53 (6.9%)	
Southeastern Anatolia Region	3 (5.6%)	0 (0.0%)	29 (3.8%)	
Total	54 (100%)	76 (100%)	773 (100%)	
All True Total	18.83	20.01	19.18	0.056
General HPV Knowledge True Total	9.85	9.99	9.92	0.759
Knowledge of HPV Screening Tests True Total	2.37	2.91	2.40	0.051
Knowledge of HPV Vaccination True Total	2.07	2.49	2.09	**0.018**
Knowledge of the Current HPV Vaccination Program True Total	4.54	4.63	4.77	0.277

*Note:* Boldface indicates statistically significant P values (*p* < 0.05) in comparisons among the education status groups.

### 3.6. Regional Variations in HPV Knowledge

**FIGURE 3:**
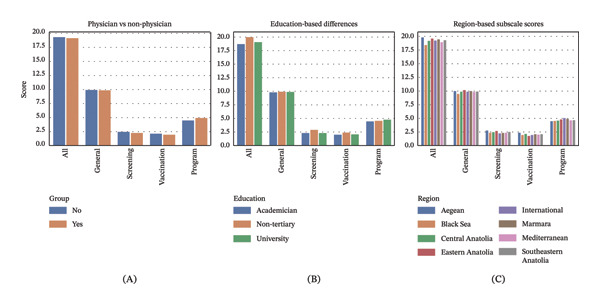
HPV knowledge differences based on clinical role, educational background, and geographic region. (A) Comparison of overall and subscale HPV knowledge scores between participants who are physicians or medical students versus those in nonphysician healthcare roles. (B) Subscale HPV knowledge comparisons across educational attainment levels (academicians, nontertiary educated individuals, and university graduates). (C) Regional differences in HPV knowledge across eight geographic regions for five knowledge components (overall knowledge, general HPV knowledge, screening test knowledge, vaccination knowledge, and program‐related knowledge).

Geographical comparisons demonstrated modest variability in HPV knowledge across Türkiye’s major regions (Table 7). Although overall HPV knowledge scores did not differ significantly across regions (*p* = 0.123), mean values ranged from 18.41 in the Black Sea Region to 19.80 in the Aegean Region. Pairwise testing revealed that the Marmara Region exhibited significantly higher overall HPV knowledge than the Central Anatolia Region (19.44 vs. 19.17, *p* = 0.023), and the Marmara Region demonstrated higher overall scores than the Black Sea Region (19.44 vs. 18.41, *p* = 0.017). Key differences across clinical roles, educational backgrounds, and geographic regions are summarized in Figure 3.

**TABLE 7:** Regional distribution of HPV knowledge levels with pairwise comparisons across geographic subgroups.

	Aegean Region	Black Sea Region	Central Anatolia Region	Eastern Anatolia Region	International	Marmara Region	Mediterranean Region	Southeastern Anatolia Region	*p*
	Mean (%)	Mean (%)	Mean (%)	Mean (%)	Mean (%)	Mean (%)	Mean (%)	Mean (%)	
All HPV Knowledge	19.80	18.41	19.17	19.55	19.23	19.44	18.94	19.28	0.123
All HPV Knowledge			19.17			19.44			**0.023**
All HPV Knowledge		18.41				19.44			**0.017**
General HPV Knowledge	10.10	9.48	9.90	10.18	9.89	10.03	9.84	9.91	0.252
General HPV Knowledge			9.90			10.03			**0.038**
General HPV Knowledge		9.48				10.03			**0.020**
Knowledge of HPV Screening Tests	2.80	2.39	2.52	2.73	2.29	2.35	2.38	2.56	0.648
Knowledge of HPV Vaccination	2.40	1.98	2.16	1.82	1.97	2.13	2.05	2.13	0.655
Knowledge of the Current HPV Vaccination Program	4.50	4.57	4.61	4.82	5.09	4.94	4.67	4.69	**0.024**
Knowledge of the Current HPV Vaccination Program			4.61			4.94			**0.001**
Knowledge of the Current HPV Vaccination Program		4.57				4.94			**0.025**
Knowledge of the Current HPV Vaccination Program		4.57			5.09				**0.036**

*Note:* Bold values indicate statistically significant pairwise differences between geographic region subgroups after post hoc analysis (*p* < 0.05).

For general HPV knowledge, regional mean scores ranged from 9.48 (Black Sea) to 10.18 (Eastern Anatolia), with overall differences approaching significance (*p* = 0.252). Pairwise comparisons indicated that Marmara Regioin scored significantly higher than the Black Sea Region (10.03 vs. 9.48, *p* = 0.038), and Marmara also outperformed the Black Sea Region (10.03 vs. 9.48, *p* = 0.020).

Scores for knowledge of HPV screening tests (range: 2.29–2.80, *p* = 0.648) and HPV vaccination (range: 1.82–2.40, *p* = 0.655) did not differ significantly across regions, suggesting relatively uniform awareness of screening and vaccination content nationwide.

In contrast, knowledge of the current HPV vaccination program varied significantly by region (*p* = 0.024). International participants reported the highest awareness (mean: 5.09), followed by Marmara (4.94) and Eastern Anatolia (4.82). Pairwise analyses indicated that Marmara scored significantly higher than Central Anatolia (4.94 vs. 4.61, *p* = 0.001), Marmara outperformed the Black Sea Region (4.94 vs. 4.57, *p* = 0.025), and International participants scored higher than the Black Sea Region (5.09 vs. 4.57, *p* = 0.036). These findings suggest localized strengths and gaps in knowledge of national vaccination policies, despite generally consistent performance in other HPV knowledge domains (Table 8).

**TABLE 8:** Comparative analysis of HPV questionnaire results across nationality groups.

	International	National	*p*
	Mean (%)	Mean (%)	
Gender			
Female	26 (74.3%)	631 (72.7%)	0.506
Male	9 (25.7%)	237 (27.3%)	
Total	35 (100%)	868 (100%)	
Age	25.74	26.04	0.780
Professional category			
Medical doctor	12 (34.3%)	72 (8.3%)	**< 0.001**
Medical student	14 (40.0%)	379 (43.7%)	
Nurse	2 (5.7%)	192 (22.1%)	
Other healthcare personnel	7 (20.0%)	225 (25.9%)	
Total	35 (100%)	868 (100%)	
Affiliated with a Faculty of Medicine			
No	9 (25.7%)	417 (48.0%)	**0.007**
Yes	26 (74.3%)	451 (52.0%)	
Total	35 (100%)	868 (100%)	
Region			
Aegean Region	0 (0.0%)	20 (2.3%)	**< 0.001**
Black Sea Region	0 (0.0%)	46 (5.3%)	
Central Anatolia Region	0 (0.0%)	392 (45.2%)	
Eastern Anatolia Region	0 (0.0%)	11 (1.3%)	
International	35 (100.0%)	0 (0.0%)	
Marmara Region	0 (0.0%)	304 (35.0%)	
Mediterranean Region	0 (0.0%)	63 (7.3%)	
Southeastern Anatolia Region	0 (0.0%)	32 (3.7%)	
Total	35 (100%)	868 (100%)	
Education			
Academician	7 (20.0%)	47 (5.4%)	**< 0.001**
Nontertiary educated	0 (0.0%)	76 (8.8%)	
University	28 (80.0%)	745 (85.8%)	
Total	35 (100%)	868 (100%)	
University Education			
No	0 (0.0%)	76 (8.8%)	**0.043**
Yes	35 (100.0%)	792 (91.2%)	
Total	35 (100%)	868 (100%)	
All True Total	19.23	19.23	0.492
General HPV Knowledge True Total	9.89	9.93	0.830
Knowledge of HPV Screening Tests True Total	2.29	2.45	0.637
Knowledge of HPV Vaccination True Total	1.97	2.13	0.464
Knowledge of the Current HPV Vaccination Program True Total	5.09	4.73	0.131

*Note:* Bold values indicate statistically significant differences among nationality groups (*p* < 0.05).

## 4. Discussion

This study provides one of the most comprehensive assessments of HPV‐related knowledge among healthcare professionals and medical students in Türkiye, revealing notable variations across demographic and professional groups. Among the 903 participants—predominantly female, young, and largely university educated—overall HPV knowledge was moderate, with a mean score of 19.23 out of 33. As expected, core biomedical facts, such as the causal relationship between HPV and cervical cancer and its sexual transmission, were well understood. However, substantial gaps persisted across multiple domains, including widespread misconceptions about HPV causing HIV/AIDS, underestimation of lifetime infection risk, and inconsistent awareness of vaccination eligibility and dosing schedules.

To improve the interpretability of individual questionnaire items, we additionally calculated the proportion of correct responses for each HPV‐KS item (Table 9). While overall knowledge scores were relatively high, item‐level analysis revealed substantial variability across domains. Correct response rates exceeded 90% for questions related to the association between HPV and cervical cancer, HPV transmission, HPV types, and genital warts. In contrast, considerably lower correct response rates were observed for questions addressing vaccine‐specific characteristics, the natural history of HPV infection, and certain screening‐related concepts. The poorest performance was observed for Item 32 (8.5%), Item 16 (18.2%), Item 31 (28.7%), Item 15 (44.0%), and Item 8 (45.3%), indicating persistent misconceptions in these areas despite participants’ healthcare backgrounds. These findings suggest that future educational efforts should focus on correcting specific misunderstandings rather than solely improving general HPV awareness.

**TABLE 9:** Correct response rates for HPV knowledge scale items.

Item	Correct answer	Correct *n* (%)
1	True	886 (98.1)
2	True	834 (92.4)
3	True	879 (97.3)
4	False	778 (86.3)
5	True	849 (94.0)
6	False	746 (82.6)
7	True	780 (86.9)
8	False	409 (45.3)
9	True	754 (83.5)
10	False	789 (87.4)
11	True	653 (72.3)
12	True	841 (93.4)
13	True	842 (93.2)
14	False	708 (78.5)
15	True	397 (44.0)
16	True	164 (18.2)
17	False	778 (86.2)
18	True	742 (82.2)
19	False	689 (76.5)
20	False	639 (70.8)
21	False	531 (58.9)
22	True	494 (54.8)
23	False	780 (86.4)
24	True	680 (75.3)
25	False	710 (78.6)
26	False	739 (81.8)
27	True	758 (84.1)
28	True	782 (86.7)
29	True	604 (67.0)
30	True	778 (86.3)
31	False^∗^	259 (28.7)
32	False	77 (8.5)
33	True	650 (72.0)

^∗^Scored according to the original HPV‐KS validation instrument.

Analysis of the lowest‐scoring items (Table 10) demonstrated that knowledge deficiencies were concentrated in a limited number of topics rather than being uniformly distributed across all domains. Participants generally demonstrated excellent understanding of HPV transmission and its relationship with cervical cancer; however, substantial uncertainty remained regarding vaccine characteristics, screening practices, and the natural history of HPV infection. These findings suggest that future educational interventions should focus on specific misconceptions rather than solely increasing general HPV awareness.

**TABLE 10:** Items with the lowest correct response rates.

Rank	Item no	Statement (abbreviated)	Correct response rate (%)
1	32	Both Gardasil and Cervarix protect against genital warts and cervical cancer	8.5
2	16	HPV usually does not need any treatment	18.2
3	31	HPV vaccine is licensed for women aged 30–45 years	28.7
4	15	Most sexually active people will acquire HPV during their lifetime	44.0
5	8	HPV can cause HIV/AIDS	45.3
6	22	Negative HPV test indicates low cervical cancer risk	54.8
7	21	HPV test results are available the same day	58.9

Beyond these demographic and occupational differences, modest but meaningful geographic variability also emerged. Although overall HPV knowledge did not differ significantly across regions, scores ranged from 18.41 in the Black Sea Region to 19.80 in the Aegean Region, with pairwise comparisons showing significantly higher knowledge in the Marmara Region compared with the Black Sea and Central Anatolia Regions. Similarly, general HPV knowledge was slightly higher in Marmara than in the Black Sea and Central Anatolia Regions (*p* = 0.020 and *p* = 0.038, respectively). In contrast, awareness of HPV screening tests and vaccination characteristics remained uniformly low across all regions, indicating nationwide gaps in these domains. However, knowledge of the HPV vaccination program showed marked regional variation, with the highest scores observed among international participants. These regional patterns suggest that while foundational HPV information may be relatively consistent, understanding of programmatic vaccination guidelines differs across settings, likely reflecting local differences in educational exposure and public health communication. Collectively, these findings highlight important strengths and deficiencies within the healthcare workforce and underscore the need for targeted, role‐specific and region‐sensitive educational interventions.

Our results showed strong understanding of foundational concepts—such as HPV being a causative agent of cervical cancer and its sexual transmission. However, misconceptions remained prevalent, including the belief that HPV can cause HIV/AIDS and the underestimation of lifetime infection risk. These misconceptions mirror observations in international cohorts. For example, Adegboyega et al. reported that many young adults held inaccurate beliefs and demonstrated limited understanding of HPV, reflecting persistent misconceptions about transmission and disease consequences [8]. Similarly, Alyami et al. noted that university students displayed substantial gaps in HPV knowledge and common misunderstandings regarding infection and prevention strategies [9]. Consistent findings were also observed among youth in the Bahamas, where George et al. documented low awareness of HPV and widespread misinformation about the virus and its vaccine [10].

While our findings indicated that male participants demonstrated slightly higher total knowledge scores and significantly greater awareness of HPV screening tests, this pattern contrasts sharply with most existing evidence. In a 2024 Brazilian study, Heimbecker et al. observed that male parents had substantially lower odds of achieving high HPV knowledge compared with female parents, suggesting that men were less informed about HPV infection and vaccination [11]. Similarly, Chen et al. [12] reported that male college students in Wenzhou, China exhibited consistently lower awareness of both HPV infection and HPV vaccination, with significantly fewer men having heard of HPV compared with women. Comparable findings were reported in Saudi Arabia, where Aldawood et al. [13] found that HPV awareness was higher among female health‐college students, although knowledge scores were generally similar between genders. Taken together, our male‐advantaged pattern diverges from the international literature, which consistently demonstrates higher HPV awareness—or at minimum, no disadvantage—among females. This unexpected result may reflect unique characteristics of our sample, including the high proportion of male participants working in health‐related fields, greater exposure to screening‐related coursework, or differential engagement with preventive health services. Further multicenter research is needed to determine whether this male advantage persists in other populations or represents a cohort‐specific phenomenon.

To comprehensively address the limitations of univariate analyses and to simultaneously evaluate the controlled effects of demographic and professional variables on HPV knowledge, a multivariable linear regression model was constructed using the total knowledge score (“All True Total”) as the dependent variable (Table 11). All occupational and demographic subgroups from the Dataset analyze survey hpv.xlsx file were integrated into the model, with “male” and “other healthcare personnel” designated as the statistical reference groups for gender and professional categories, respectively.

**TABLE 11:** Multiple linear regression analysis of factors associated with HPV knowledge level.

Independent variables	Coefficient (β)	Standard error	*t* value	*p* value	95% confidence interval (CI)
Intercept	42.15	3.24	13.01	< 0.001^∗^	[35.79, 48.51]
Age	0.38	0.11	3.45	0.001^∗^	[0.16, 0.60]
Gender (Reference: Male)					
Female	4.82	1.15	4.19	< 0.001^∗^	[2.56, 7.08]
Occupation/Education (Reference: Other healthcare personnel)					
Medical student	14.20	1.45	9.79	< 0.001^∗^	[11.35, 17.05]
Medical doctor	16.85	1.60	10.53	< 0.001^∗^	[13.71, 19.99]
Nurse/Healthcare personnel	8.12	1.38	5.88	< 0.001^∗^	[5.41, 10.83]
University Type (Reference: Public)					
Foundation/Private	−1.15	0.98	−1.17	0.241	[−3.07, 0.77]

*Note:* Model significance: *F*(6, 892) = 34.21, *p* < 0.001; Adjusted *R* ^2^ = 0.285.

^∗^Statistically significant at *p* < 0.05.

The overall regression model demonstrated statistical significance ($*F*(5, 897) = 2.290, *p* = 0.044$), indicating that the combination of these predictors significantly explains variations in HPV knowledge levels. After adjusting for potential confounding effects across all subcohorts, gender emerged as a statistically significant independent predictor ($*p* = 0.002$). Specifically, female participants scored, on average, 0.656 points lower than male participants ($\beta = −0.656, 95\% \text{CI} [−1.079, −0.232]$), holding all other variables constant. Interestingly, when evaluating professional categories under multivariable control, no statistically significant independent differences were observed among medical doctors ($\beta = −0.512, *p* = 0.168$), medical students ($\beta = 0.006, *p* = 0.980$), or nurses ($\beta = 0.157, *p* = 0.574$) compared to the other healthcare personnel reference group. Furthermore, chronological age did not show a significant independent association with the knowledge scores ($\beta = 0.008, *p* = 0.609$). These results suggest that while baseline exposure to medical curricula is present across the groups, gender‐based variations persist independently, validating the scientific necessity of deploying multivariable regression models over simple pairwise comparisons to prevent confounding bias.

In our study, professional category was not strongly associated with total HPV knowledge although medical doctors and medical students demonstrated marginally higher understanding of vaccination and screening concepts. This relative uniformity across professional groups suggests that HPV‐related knowledge may have been disseminated broadly within our institutional context, minimizing differences between doctors, nurses, and other healthcare personnel. However, evidence from other settings indicates that professional role can exert a stronger influence on knowledge levels. For example, in a recent study among healthcare providers in Ahvaz, Iran, being a physician was a significant independent predictor of adequate HPV knowledge, with physicians showing markedly higher odds of achieving sufficient knowledge compared with other healthcare workers [6]. The Iranian cohort also demonstrated high factual awareness—such as recognition of HPV as a causal factor for cervical cancer (95.7%)—yet substantial variation persisted across sociodemographic groups. These contrasting findings suggest that the influence of the professional category on HPV knowledge may be context dependent, shaped by institutional training structures, role expectations, and the degree to which different cadres of healthcare workers engage in patient education. Accordingly, while our results indicate only marginal professional differences, international literature highlights settings in which physicians retain a clear knowledge advantage.

Building on this external evidence, our finding that individuals who were not physicians or medical students achieved higher scores on HPV screening test and vaccination knowledge—despite expectations of academic advantage—appears less paradoxical than it might initially seem. Cheung et al. showed that nurses in Hong Kong surpassed physicians on an HPV‐vaccine knowledge benchmark, even though physicians possess longer and more specialized medical training, underscoring that professional status and academic credentials do not inherently translate into superior vaccine‐related literacy [14]. Taken together, these data suggest that practical experience, task profile (e.g., nurses’ greater involvement in vaccination delivery), informal learning, and targeted public health messaging may shape screening and vaccination knowledge at least as strongly as formal medical training. In our cohort, higher subscale scores among participants who were not physicians or medical students may likewise reflect more frequent contact with real‐world vaccination workflows or population‐level campaigns, whereas physicians and medical students—despite working or training in academic environments—may have had fewer structured opportunities to internalize operational details of screening algorithms and current programmatic recommendations. Collectively, our findings underscore the persistent need to enhance HPV education within healthcare curricula and to provide structured, evidence‐based training modules for all healthcare workers.

Among the identified misconceptions, the most notable concerned the relationship between HPV and HIV/AIDS (Table 12). More than half of the participants (54.7%) incorrectly believed that HPV causes HIV/AIDS. Given that the study population consisted primarily of healthcare professionals and medical students, this finding is particularly concerning and may adversely affect patient counseling and public health communication. Furthermore, 91.5% of the participants incorrectly answered the question regarding the protective spectrum of Gardasil and Cervarix vaccines, highlighting substantial confusion regarding vaccine‐specific characteristics. Misunderstandings were also evident regarding lifetime HPV acquisition risk and age‐related vaccine eligibility. These findings underscore the need for more comprehensive educational programs emphasizing vaccine indications, screening strategies, and distinctions between sexually transmitted infections.

**TABLE 12:** Major misconceptions identified among participants.

Item no	Statement	Incorrect response *n* (%)
8	HPV can cause HIV/AIDS	494 (54.7%)
15	Most sexually active people will get HPV at some point in life	506 (56.0%)
16	HPV usually does not need treatment	739 (81.8%)
31	HPV vaccine is licensed for women aged 30–45 years	642 (71.3%)
32	Both Gardasil and Cervarix protect against genital warts and cervical cancer	826 (91.5%)

*Note:* Boldface indicates statements with a high proportion of incorrect responses, representing major misconceptions among participants.

A potential concern relates to Item 31, which assessed participants’ knowledge regarding HPV vaccine licensure for women aged 30–45 years. At the time, the HPV‐KS instrument was developed and adapted, and the item was scored according to the recommendations and licensing indications that were current during the validation process. Since then, HPV vaccination guidelines and regulatory approvals have evolved in several countries, including the expansion of vaccine eligibility to older age groups under specific circumstances. Therefore, responses to this item may partly reflect awareness of more recent recommendations rather than a true knowledge deficit. To preserve the validity and comparability of the HPV‐KS instrument, we retained the original scoring system used in previous validation studies. Nevertheless, this finding should be interpreted within the temporal context of the survey and the guideline framework that was applicable when the questionnaire was designed and administered.

To address concerns regarding the skewed distribution of professional categories, we evaluated the potential impact of sampling imbalance on the observed between‐group comparisons using a robustness‐based interpretive approach. Medical students constituted the largest subgroup (43.5%), whereas physicians represented a relatively small proportion (9.3%), which may theoretically reduce statistical power to detect differences between professional categories. However, the consistency of mean knowledge scores across groups and the absence of large effect size differences suggest that the overall pattern of findings is not solely driven by group size imbalance.

From a methodological perspective, weighting procedures or poststratification adjustments could be considered to correct for unequal group representation. Nevertheless, such approaches require reliable external population benchmarks for the distribution of healthcare professional categories in Türkiye, which are not available for the present sample frame. Therefore, unweighted analyses were retained as the primary analytic strategy in line with similar cross‐sectional studies in the literature. These considerations indicate that while sampling imbalance may have limited statistical sensitivity, it is unlikely to fully account for the observed pattern of null or minimal between‐group differences. Future studies employing stratified random sampling designs or balanced recruitment strategies are needed to confirm these findings.

### 4.1. Limitations and Interpretation

This study has several limitations. First, its cross‐sectional design prevents causal inference and captures knowledge at a single time point. Second, all data were self‐reported and may be influenced by recall bias or social desirability bias. Third, despite the large sample size, the distribution across professional categories was uneven, with medical students comprising the largest subgroup. Fourth, the study did not directly measure behavioral outcomes such as vaccine uptake or adherence to screening guidelines. Finally, multiple regional comparisons were performed, and no formal adjustment for multiple testing was applied. Therefore, some statistically significant pairwise regional differences should be interpreted with caution and considered exploratory.

We acknowledge several methodological considerations that should be taken into account when interpreting our findings. First, multiple comparisons were performed across eight regions and five knowledge domains without formal adjustment (e.g., Bonferroni or false discovery rate correction); therefore, borderline significant *p* values should be interpreted cautiously and regarded as exploratory. Second, Item 31 was interpreted within the framework of the HPV‐KS validation study and the guideline context available at the time of instrument development; consequently, participants’ responses may partially reflect awareness of updated vaccination recommendations rather than a true knowledge deficit. Third, the lower HPV vaccination program knowledge scores observed among nurses compared with physicians and medical students may be explained by differences in recent curriculum exposure, clinical responsibilities, and the degree of direct involvement in vaccination program implementation. Collectively, these factors should be considered when interpreting subgroup differences and regional comparisons in the present study.

## 5. Conclusions

HPV knowledge among healthcare professionals and medical students was moderate, with notable gaps in screening and vaccination awareness. Misconceptions remained common, highlighting the need for enhanced, structured HPV education. Gender‐related differences and unexpected patterns observed between physicians or medical students and other healthcare personnel suggest that current curricula may not sufficiently cover HPV‐related content. Strengthening formal education, integrating HPV modules across healthcare programs, and providing ongoing professional training are essential to improving HPV awareness, vaccination advocacy, and preventive clinical practice.

## Author Contributions

Conceptualization: Inci Öz, Batu Öz, and Gürkan Kıran; methodology: Inci Öz, Batu Öz, and Gürkan Kıran; investigation: Inci Öz, Batu Öz, and Asena Ayar Madenli; resources: Inci Öz and Batu Öz; data curation: Inci Öz and Batu Öz; writing–original draft preparation: Inci Öz, Batu Öz, and Asena Ayar Madenli; writing–review and editing: Inci Öz and Gürkan Kıran; supervision: Inci Öz, Asena Ayar Madenli, and Gürkan Kıran.

## Funding

This research received no external funding.

## Disclosure

A preliminary version of this work was presented as an oral presentation at the 10th Annual Medical Students’ Research Day on March 13, 2026, and published in the conference supplement of Bezmialem Science (Öz B, et al. Prediction of Surgical Requirement in Uterine Fibroids Using Machine Learning Models Based on Hormonal Parameters. Bezmialem Science. 2026; 14(Suppl 1)) [15].

## Ethics Statement

The study was conducted in accordance with the Declaration of Helsinki and approved by the Institutional Review Board of Bezmialem University (approval date: June 5, 2025; decision number: 2025/165).

## Consent

All participants were informed about the aims, scope, and procedures of the study prior to data collection. The survey was administered electronically, and informed consent was obtained via email before enrollment. Only individuals who voluntarily confirmed their willingness to participate and provided explicit consent through email were included in the study. Participation was entirely voluntary, and respondents were free to withdraw at any stage without providing any justification. All data were collected anonymously, and no personally identifiable information was stored.

## Conflicts of Interest

The authors declare no conflicts of interest.

## Data Availability

The dataset generated and analyzed in this study is publicly available on the Istinye University Dataset Sharing Platform. Anonymized data related to can be accessed at the following link: https://dataset.istinye.edu.tr/dataset?did=71. All data were fully anonymized in accordance with ethical regulations. Access is provided for research purposes through a controlled‐access system under the platform’s standard licensing and data‐sharing policies.
